# Mining the *Utricularia gibba* genome for insulator-like elements for genetic engineering

**DOI:** 10.3389/fpls.2023.1279231

**Published:** 2023-11-08

**Authors:** Daniel Laspisa, Eudald Illa-Berenguer, Sohyun Bang, Robert J. Schmitz, Wayne Parrott, Jason Wallace

**Affiliations:** ^1^ Center for Applied Genetic Technologies, University of Georgia, Athens, GA, United States; ^2^ Institute of Bioinformatics, University of Georgia, Athens, GA, United States; ^3^ Department of Genetics, University of Georgia, Athens, GA, United States; ^4^ Department of Crop & Soil Science & Institute of Plant Breeding, Genetics and Genomics, University of Georgia, Athens, GA, United States

**Keywords:** bladderwort, insulator, transgenics, *Utricularia*, *cis*-regulatory elements

## Abstract

**Introduction:**

Gene expression is often controlled via cis-regulatory elements (CREs) that modulate the production of transcripts. For multi-gene genetic engineering and synthetic biology, precise control of transcription is crucial, both to insulate the transgenes from unwanted native regulation and to prevent readthrough or cross-regulation of transgenes within a multi-gene cassette. To prevent this activity, insulator-like elements, more properly referred to as transcriptional blockers, could be inserted to separate the transgenes so that they are independently regulated. However, only a few validated insulator-like elements are available for plants, and they tend to be larger than ideal.

**Methods:**

To identify additional potential insulator-like sequences, we conducted a genome-wide analysis of *Utricularia gibba* (humped bladderwort), one of the smallest known plant genomes, with genes that are naturally close together. The 10 best insulator-like candidates were evaluated in vivo for insulator-like activity.

**Results:**

We identified a total of 4,656 intergenic regions with expression profiles suggesting insulator-like activity. Comparisons of these regions across 45 other plant species (representing Monocots, Asterids, and Rosids) show low levels of syntenic conservation of these regions. Genome-wide analysis of unmethylated regions (UMRs) indicates ~87% of the targeted regions are unmethylated; however, interpretation of this is complicated because *U. gibba* has remarkably low levels of methylation across the genome, so that large UMRs frequently extend over multiple genes and intergenic spaces. We also could not identify any conserved motifs among our selected intergenic regions or shared with existing insulator-like elements for plants. Despite this lack of conservation, however, testing of 10 selected intergenic regions for insulator-like activity found two elements on par with a previously published element (EXOB) while being significantly smaller.

**Discussion:**

Given the small number of insulator-like elements currently available for plants, our results make a significant addition to available tools. The high hit rate (2 out of 10) also implies that more useful sequences are likely present in our selected intergenic regions; additional validation work will be required to identify which will be most useful for plant genetic engineering.

## Introduction

Modern crop improvement frequently stacks multiple transgenes into a single cultivar. The percentage of stacked-gene transgenic cultivars grown in the United States has increased from ~52% in 2012 to ~81% in 2022 (Dodson, 2022). Currently, these are deployed as breeding stacks, created by individually transforming the genes, and then crossing the resulting plants together and looking for the desired segregants (Steiner et al., 2013). As the number of independently segregating genes increases, the process becomes unwieldy. Hence molecular stacks – which contain many genes in one construct – are becoming attractive, as they segregate as a single Mendelian unit.

Molecular stacks present a challenge, as cross-regulation within transgenic cassettes can result in altered or unintended expression of the transgenes (Singer et al., 2012). A way to address these issues in non-plant organisms is to include transcriptional blockers in transgenic constructs which serve to compartmentalize a cassette by limiting the effect of regulators related to proximal genes (Padidam and Cao, 2001; Yang et al., 2011; Singer et al., 2012). For plants, such transcriptional blockers are often called insulator-like elements or simply insulators when used in transgene cassettes. “Insulators” in the context of plant genetic engineering are distinct from the genomic insulators that separate chromatin compartments in animal genomes (Heger and Wiehe, 2014; Matzat and Lei, 2014).

Canonical genomic insulators as observed in animals regulate expression through enhancer-blocking, and barrier functions (Scott et al., 1999; Wang et al., 2014). Pairs of genomic insulators have been shown to interact forming topologically associated domains (Fujioka et al., 2016). To date, no such genomic insulators comparable to those from animals have been confirmed in plants, although evolutionarily conserved genome structure and expression patterns may suggest their existence (Rowley et al., 2017; Kurbidaeva and Purugganan, 2021).

The lack of any confirmed functional conservation with insulators in animals makes sequences with insulator function in plants challenging to identify. Over the last two decades, only eight such sequences have been identified (Hily et al., 2009; Yang et al., 2011; Zhang et al., 2012; Singer and Cox, 2013; Liu et al., 2022; Illa-Berenguer et al.).

The goal of this project was to identify additional sequences that can function as insulator-like elements for genetic engineering in plants. In non-plant organisms, many different techniques can be used to identify novel CREs in genomes, including ATAC-seq for open chromatin (Maher et al., 2018; Ricci et al., 2019; Marand et al., 2021), Hi-C or similar technology for chromatin conformation (Dixon et al., 2012; Van Bortle et al., 2014), STARR-seq for active transcription (Arnold et al., 2013; Ricci et al., 2019; Jores et al., 2020; Tian et al., 2022), and bisulfite sequencing for DNA methylation (Hou et al., 2012; Liu et al., 2017). However, given the lack of evidence that plant insulators function in the same manner as animal insulators, along with the lack of evidence that sequences with insulator function in transgenic constructs function as such *in-situ*, our search focused on conserved gene pairs with different expression levels.

We selected the *Utricularia gibba* (humped bladderwort) genome to mine for potential regulatory elements for genetic engineering. *U. gibba* belongs to the *Lentibulariaceae* family of carnivorous plants, and it is found in aquatic environments and waterlogged soil across all continents but Antarctica (Duenas, 2022). We chose it for this analysis because it has one of the smallest sequenced plant genomes to date (~82-100 Mb) while still retaining the typical number of genes for a flowering plant (~30,000) (Lan et al., 2017). Phylogeny-based analysis of genome size evolution in the *Lentibulariaceae* suggests that *U. gibba*’s small genome is a result of reduction through deletions of redundant genes and repression/deletion of transposable elements (Ibarra-Laclette et al., 2013; Fleischmann et al., 2014; Carretero-Paulet et al., 2015). Remarkably, *U. gibba* has undergone at least three whole genome duplication events in its evolutionary history, but the expansion was balanced by high rates of gene fractionation (Ibarra-Laclette et al., 2013; Carretero-Paulet et al., 2015). The deletions reduced the genome down to less than 10% of an estimated ancestral size of ~1.5 Gb (Veleba et al., 2014). These mechanisms resulted in a highly compact and shuffled genome (Ibarra-Laclette et al., 2013). Furthermore, the deletion of superfluous genomic DNA while maintaining a typical number of genes resulted in short intergenic regions that are ideal for mining for potential novel elements. The small size of *U. gibba*’s intergenic spaces reduces the search space and means that initial hits are likely to be relatively short, making them particularly useful for genetic engineering. Although various methods exist to transform large constructs into plants (Van Eck et al., 1995; Hamilton et al., 1996; Shibata and Liu, 2000), shorter elements are easier to work with and tend to transform at higher efficiency.

We previously showed that comparisons of expression level and orientation of gene pairs could identify intergenic regions with insulator-like activity, resulting in three sequences (Ugi1, Ugi3, and Ugi4) that performed as well as or better than published insulator-like sequences (Illa-Berenguer et al.). The current work builds on these results by using additional RNAseq data, bisulfite sequencing data, and an expanded synteny analysis across 45 angiosperm genomes to better identify insulator-like sequences in *U. gibba*.

## Results

### Distribution and characteristics of potential insulator-like elements in *Utricularia gibba*

**Figure 1:**
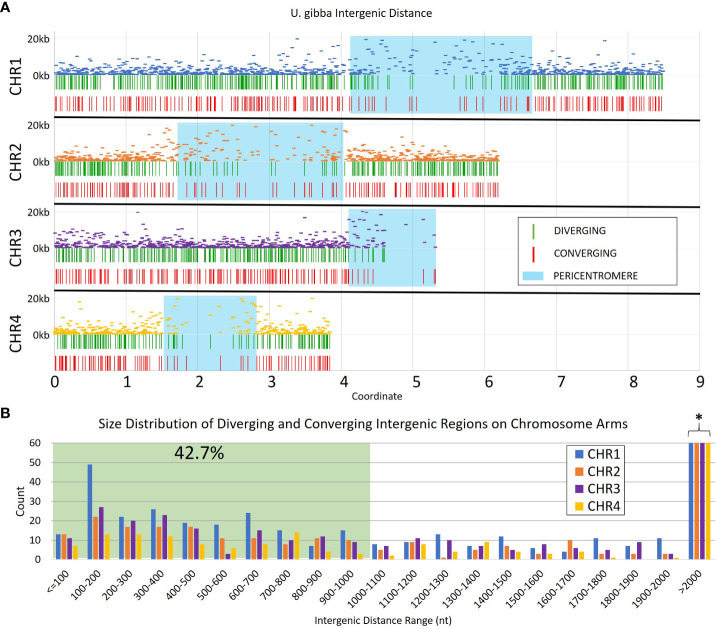
Intergenic distances in assembled *Utricularia gibba* chromosomes 1-4. **(A)** The relative intergenic distances normalized to the maximum (20 kb) for pairs of annotated genes on chromosomes 1-4, and the distribution of convergent and divergent potential insulators across the chromosomes. Approximate pericentromeric regions are shaded in blue and were defined by the primary cluster of repeats in the 4 chromosomes (**Supplementary Figure 1**). **(B)** Intergenic regions with lengths >1 kb were filtered from the dataset to focus on shorter elements (green shading) that could be more easily used in transgenic constructs. The resulting filtered dataset represents roughly 42.7% of all intergenic regions in *U. gibba*. The full count for regions >2000 nt is cut off for readability (marked by *), and is 192, 139, 112, and 87 for CHR1-CHR4, respectively (see **Supplementary Table ST1**).

We generated a set of 30,756 transcripts using Cufflinks (Trapnell et al., 2012) from publicly available RNAseq data (NCBI bioprojects PRJNA595351 and PRJNA354080); this number is consistent with the 30,689 gene models reported in the long-read assembled genome of *U. gibba* (Lan et al., 2017). These gene models were used to identify the portion of the genome corresponding to intergenic space (**Figure 1**). From our annotations, we estimate that ~48% of the *U. gibba* genome is genic, which is consistent with a previously published estimate of ~51% (Ibarra-Laclette et al., 2013). The lengths of the intergenic regions were limited to a range of 50 nt to 20 kb, which is enough to accommodate ~2 transposable element insertions (Wells and Feschotte, 2020). The largest intergenic distances are located in the pericentromeric regions, where transposons normally accumulate and where MUMmer (Kurtz et al., 2004) identified a high density of repetitive sequences (**Supplementary Figure 1**), although chromosome 4 showed reduced repeat density relative to the other three chromosomes.

**Figure 2:**
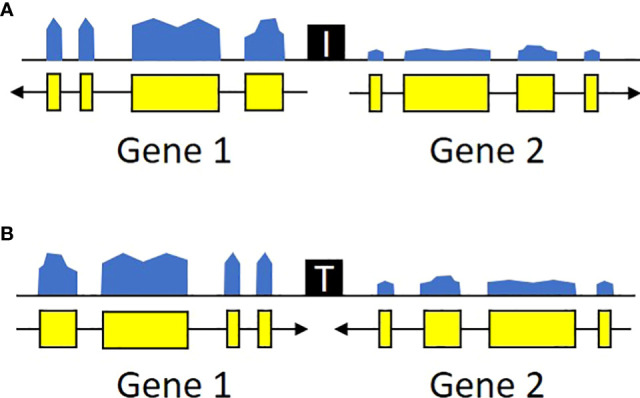
Transcriptional profiles of potential insulators. *U. gibba* intergenic regions were classified as potential insulators based on the local orientation and expression levels of the flanking genes. Specifically, we targeted regions where the flanking genes were either **(A)** diverging or **(B)** converging and had expression levels differing by at least 1.5x. These regions also likely contain additional *cis*-regulatory elements (promoters, terminators, etc.) outside the scope of this study. Ultimately, these criteria were chosen to enrich for candidates with possible insulator function instead of exhaustive catalog of all sequences with any potential regulatory function.

We focused on intergenic spaces (regions between the termini of predicted gene models) where the neighboring genes were in opposite orientations and had a minimum of 1.5-fold change difference between the gene pairs. The loose fold change threshold ensured a high number of hits prior to filtering. These criteria were chosen to enrich for potential insulator-like activity, under the hypothesis that sequences that prevent one gene’s transcription from activating its neighbor are most easily found between genes with different expression levels, and that genes with opposite orientations are less likely to contain transcriptional terminators. We identified a total of 4,656 intergenic regions that met these criteria, 2,658 with divergent orientations (head-to-head, so their 5’ ends are adjacent and transcription proceeds away from each other) and 1,998 with convergent ones (tail-to-tail, so the 3’ ends are adjacent and transcription is toward each other) (**Figure 2**, **Supplementary Table 1**). These regions are roughly randomly distributed across the *U. gibba* chromosomes (**Figure 1**). We then focused on intergenic regions shorter than 1 kb because these would be the most useful for genetic engineering applications. This length threshold limits our targets to ~42.7% of the total identified intergenic regions. This filter also removed more intergenic regions near the centromeres, probably due to higher transposon presence (Cervantes-Pérez et al., 2021) making these regions longer.

### Targeted intergenic regions are generally in unmethylated regions of the chromosome

Unmethylated regions (UMRs) coincide with the majority of CREs in typical plant genomes (Zhang et al., 2006). Previous analyses of DNA methylation in *U. gibba* found reduced global DNA methylation compared to *Glycine max* and *Arabidopsis thaliana* (Cervantes-Pérez et al., 2021), so we expected that there could be considerable overlap between potential CREs and UMRs in the *U. gibba* genome. DNA methylation patterns around the targeted intergenic regions were investigated using previously published whole-genome bisulfite sequencing data (NCBI accession PRJNA633566) (Cervantes-Pérez et al., 2021). DNA methylation was generally low across the genome, with a mCG fraction of ~10% compared to a reported 24% in *A. thaliana* and 51% in *G. max* (Cokus et al., 2008; Schmitz et al., 2011; Schmitz et al., 2013). The mCHG and mCHH fractions in *U. gibba* (~8% and ~6%, respectively) are slightly elevated compared to those in *Arabidopsis* (mCHG ~7% and mCHH ~2%). However, the global methylated fraction of *U. gibba* (~24%) is reduced relative to that of *Arabidopsis* (~32%). The highest levels of DNA methylation occur in the centromeres and pericentromeres of each chromosome (**Supplementary Figure 2**). Previous investigations of methylation patterns in *U. gibba* had attributed globally reduced levels of methylation to truncated or missing RNA-directed DNA methylation (RdDM) proteins (Cervantes-Pérez et al., 2021).

Although global methylation was lower than observed in *Arabidopsis*, we were still able to identify unmethylated regions (UMRs) based on local decreases in DNA methylation of one or more gene contexts. We found decreases in DNA methylation levels for all three contexts (CG, CHG, and CHH) over our targeted intergenic regions, with 4,046 of 4,656 overlapping with an identified UMR (~86.9%). These UMRs are generally larger than the intergenic regions (mean length of 9.8 kb for UMRs vs 2.4 kb for intergenic regions), which could be expected given the low frequency of repetitive DNA in *U. gibba* (**Supplementary Figure 1**). In genomes with high repeat content, certain repetitive elements can target gene proximal regions and potentially isolate regulatory elements in intergenic regions over time (Jiang and Wessler, 2001; Gao et al., 2012). Some repeat elements rapidly become methylated, while CREs generally remain unmethylated allowing the isolation of individual CREs (Sigman and Slotkin, 2016; Crisp et al., 2020; Ramakrishnan et al., 2021). However, the low global levels of methylation in *U. gibba* result in large predicted UMRs, precluding the use of methylation to efficiently refine CRE boundaries.

### Limited conservation of *Utricularia gibba* synteny in monocots, asterids, and rosids

**Figure 3:**
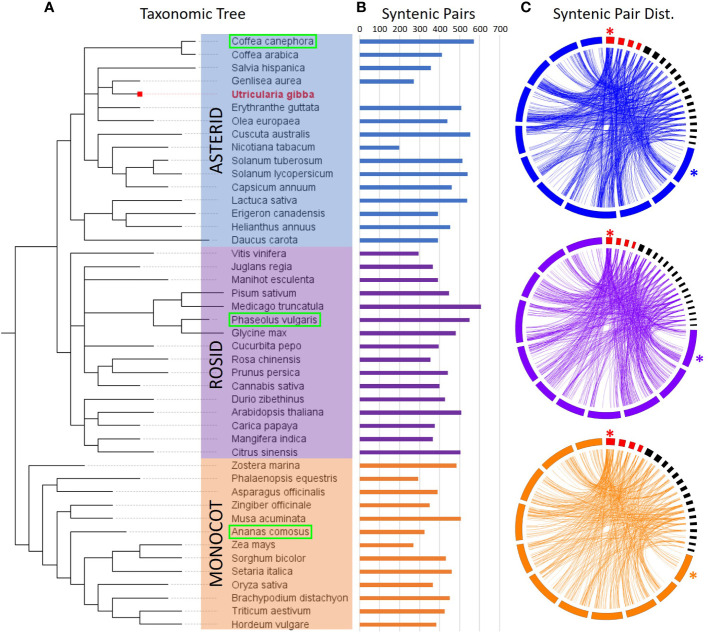
Synteny of CREs across 45 physical maps (including *U. gibba*). **(A)** Taxonomic arrangement of the 45 species investigated for syntenic relationships with *U. gibba*, based on NCBI taxonomy. The 45 species fall into three primary groups: the Asterids (blue, including *U. gibba*), Rosids (purple), and Monocots (orange). **(B)** The number of total syntenic gene pairs with *U. gibba* is variable within each of the three groups, though the outgroup (Monocots) is generally less syntenic with *U. gibba.* **(C)** Circos (Krzywinski et al., 2009) synteny plots for one representative genome from each group (boxed in green in Panel **A**). *U. gibba* assembled chromosomes are shown in red and scaffolds >1 Mb in black, with chromosome 1 in each assembly marked with an asterisk. Chromosomes are displayed clockwise in ascending order. Synteny between *U. gibba* and other genomes is generally low due to the high level of fractionation previously described in *U. gibba* (Ibarra-Laclette et al., 2013).

To enrich our dataset for conserved sequences, we looked at the evolutionary conservation of regions. Unfortunately, multiple rounds of whole-genome duplications followed by fractionation during *U. gibba* evolutionary history have rendered macro syntenic investigations impractical (**Supplementary Figure 3**). Instead, we used microsynteny to evaluate the conservation of these regions (Ibarra-Laclette et al., 2013; Carretero-Paulet et al., 2015). We investigated a set of 45 physical maps comprising 16 asterids, 16 rosids, and 13 monocots, each representing increasing evolutionary distance from *U. gibba*. (**Figure 3**, **Supplementary Table 1**). Microsyntenic regions were defined as gene pairs where a) both *U. gibba* genes have a BLASTP hit (minimum evalue 1e-5) in the non-*U. gibba* physical map, and b) there are ≤5 genes between the gene pairs in both the *U. gibba* and orthologous genome. As expected, we observe slightly fewer syntenic gene pairs between *U. gibba* and monocots due to increased divergence time relative to the other two lineages. We also observe considerable variability in syntenic gene pairs within the lineages, which is likely the result of a combination of factors, such as evolutionary history and the assembly quality of the non-*U. gibba* genome. By these criteria, we found 1,299 of our intergenic regions that are present in syntenic positions in at least one other non-*U. gibba* physical map (**Supplementary Table 2**). Of these, 37 (2.8%) were considered “high confidence”, meaning the sequences were ≤1 kb in length, there was > 10-fold change in gene expression between the genes in *U. gibba*, and both the orientation and number of gene models in *U. gibba* was conserved in at least one orthologous species.

### Motif identification among selected intergenic regions

**Figure 4:**
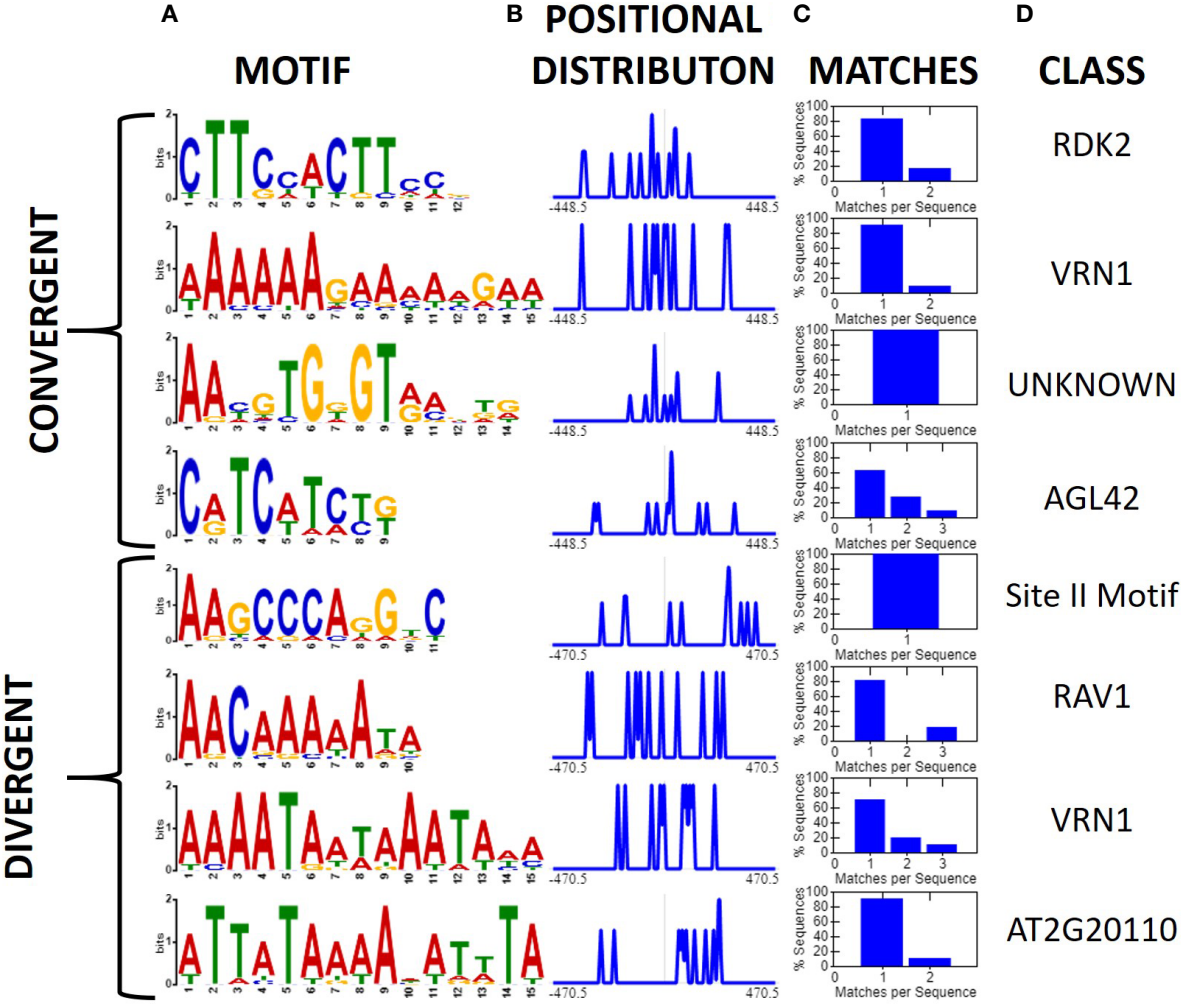
Motifs associated with high confidence convergent and divergent intergenic regions (XTREME, Bailey et al., 2015). High confidence regions were those ≤1 kb in length, >10-fold expression difference between genes, and sharing the orientation and order of genes in at least one other genome. **(A)** Conserved motifs among 17 divergent, and 20 convergent high confidence intergenic regions displayed as a sequence logo, **(B)** positional distribution relative to the middle of the intergenic sequence, and normalized to the maximum, **(C)** Histogram displaying the number of query sequences containing the motif and instances per sequence. **(D)** The putative class of transcription factor or binding site associated with the motif.

Initially, we investigated all convergent and divergent regions ≤ 1 kb using XSTREME (Bailey et al., 2015). A total of 12 motifs were found to be shared between two or more expression patterns. Only one of these motifs, a CCT short tandem repeat motif, was shared between convergent and divergent regions (**Supplemental file Image 1.PNG**). Several promoter associated motifs were also uncovered which was not unexpected as our classification schemes inherently contain the promoters for both genes in regions flanked by diverging genes (**Figure 2**). After filtering high confidence intergenic regions (i.e., those ≤1 kb, >10-fold expression difference (FPKM) between genes flanking the intergenic region, and the sequence was shared by at least one other physical map), we investigated them for conserved motifs (**Figure 4**). Divergent intergenic regions contained three A-T rich motifs and the promoter associated Site II motif. The convergent intergenic regions contained RDK2 and AGL42 motifs as well as a motif that was not associated with any identified binding site. Only one of the eight identified motifs, an A-T rich VRN1 related motif, was shared between the divergent and convergent groups.

### *In vivo* evaluation of insulator-like activity

From our list of intergenic regions, we selected 18 of the strongest candidates to directly test for insulator-like activity in a transgenic context. Nine of these sequences were identified based on high fold change expression differences in their neighboring genes, while the other nine were identified as those called as syntenic with the largest number of genomes, regardless of fold change (see methods for details).

**Figure 5:**
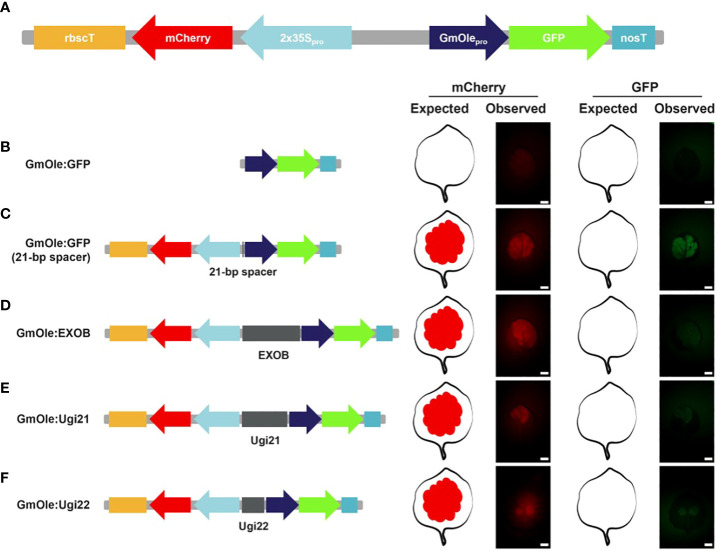
Dual reporter assay to evaluate the potential insulator activity in *Nicotiana benthamiana* leaves. **(A)** Schematic representation of the dual reporter assay. The fluorescent reporters are mCherry driven by the 2x35S promoter and GFP under the soybean seed-specific oleosin (GmOle) promoter. **(B)** When GmOle : GFP is transfected into *N. benthamiana* leaves, there is no GFP fluorescence present. **(C)** But, when the two cassettes are linked together by a 21-bp spacer, the enhancer from the 2x35S promoter ectopically activates the GmOle : GFP construct in leaf tissue. **(D)** However ectopic GFP expression is significantly attenuated when EXOB, a sequence known to have insulator function (Singer et al., 2010) is used to link the two cassettes. **(E, F)** Similarly, the 2 bladderwort sequences identified (Ugi21 or Ugi22) also attenuate ectopic GFP expression.

All the selected sequences (**Supplementary Table 1**) were ordered as DNA fragments. Four of them could not be synthesized due to either high GC content (Ugi23 and Ugi33) or poly(A/T) repeats (Ugi27) or did not pass the manufacturer quality control specifications (Ugi37). Ugi24 was not ordered because it contains a *Bsa*I restriction site, which cannot be properly cloned using the GreenGate system. Out of the 13 sequences that could be synthesized, three could not be cloned into entry vectors despite multiple attempts. Of the 10 remaining intergenic regions, – four selected based on fold change expression differences and six based on the microsyntheny criteria – were cloned and evaluated in an *in vivo* dual-reporter assay, following the protocol of (Illa-Berenguer et al.) (**Figure 5**). In brief, elements to test were placed between an mCherry reporter driven by the 2x35s constitutive promoter and, in the opposite (diverging) direction, GFP driven by the seed-specific oleosin promoter. By itself, the oleosin promoter is not expressed in leaf tissues, but it can be activated by the enhancer element found within the adjacent 2x35S promoter (Benfey et al., 1990). Inserts with insulator-like activity reduce GFP fluorescence relative to the negative control (a 21-bp spacer); the lower the GFP readout, the stronger the insulator. Note that simply putting both genes next to each other also slightly decreases expression of mCherry. Such interference between gene pairs has long been observed in plants (Padidam and Cao, 2001) and animals (Eszterhas et al., 2002).

**Figure 6:**
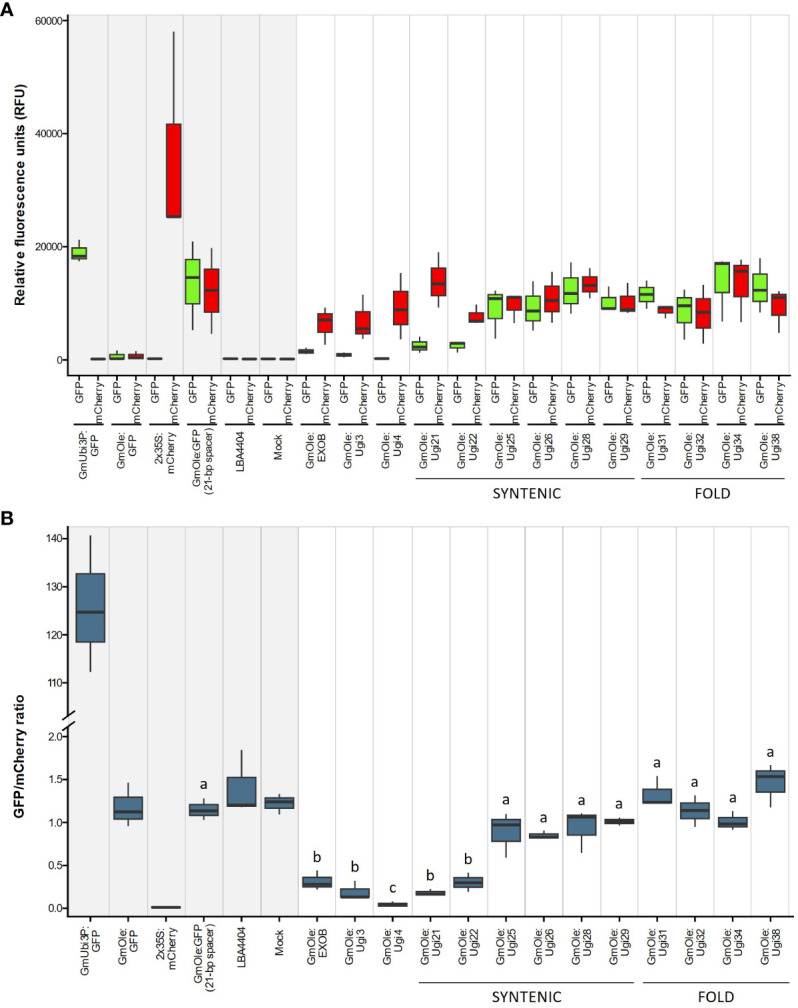
Evaluation of putative insulator activity **(A)** Red and green represents expression of mCherry (driven by the 2x 35S promoter) and GFP (driven by either the constitutive GmUbi3 promoter or the seed-specific *Glycine max* oleosin (GmOle promoter). Columns with a gray shaded background represent the controls (expression cassettes containing only mCherry or GFP by themselves, dual-cassette with a 21-bp spacer sequence, *Agrobacterium* (strain LBA4404) with no binary plasmid, and infiltration buffer). Columns with a white background represent expression of mCherry and GFP cassettes linked together in one construct. Sequences being tested for insulator activity were placed between the fluorescent markers, and GFP expression is attenuated if there is an insulator effect. The first set of columns shows sequences previously verified to have an insulator effect [EXOB (Singer et al., 2010), Ugi3, and Ugi4 (Illa-Berenguer et al.)]. The remaining columns show potential insulators from this study, identified either by fold-expression change of adjacent gene pairs or through shared synteny with multiple genomes Notice that 21-bp spacer sequence between the two cassettes does not affect expression of GFP, showing that separating the two cassettes is not enough to stop GFP activation. Three biological replicates were performed for each construct. **(B)** The results from **Figure 6A** expressed in terms of the ratio of mCherry expression to GFP expression. Sequences showing a significantly lower ratio are those with insulator activity. Different letters above boxplots indicate significantly different groups as determined by one-way ANOVA followed by Tukey-HSD *post-hoc* test (α ≤ 0.05). The y-axis was broken between 2 and 100 to better show the higher values.

**Figure 7:**
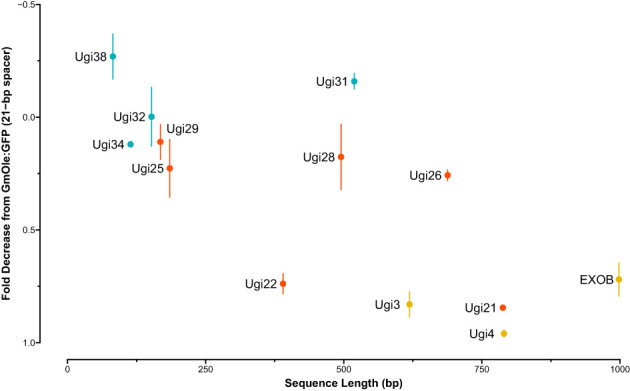
Insulator strength vs sequence length. The sequences tested for insulator-like activity are shown with size (bp) on the x-axis and fold-change decrease in fluorescence signal relative to control (dual-cassette with a 21-bp spacer sequence) on the y axis. Fold-change and syntenic insulator sequences are shown in blue and red, respectively. Previously reported sequences with insulator function from EXOB (Singer et al., 2010) and (Illa-Berenguer et al.) (Ugi3 and Ugi4) are in yellow. The largest, least effective sequences in this test system are in the upper right, while the smallest, most effective are in the lower left.

The dual-reporter assay showed that two sequences, Ugi21 and Ugi22, have insulator-like activity (**Figure 6**). These insulators reduced GFP expression to 15.5 and 26.1% on average, respectively, compared to the 21-bp spacer control and are comparable with the effect of the existing EXOB insulator (Singer et al., 2010) (**Figure 7**), which is the best of the non*-Utricularia* insulators tested (Illa-Berenguer et al.). Those values are also comparable to Ugi3 insulator activity (Illa-Berenguer et al.). Ugi21 and Ugi22 are 212 nt and 610 nt shorter than EXOB respectively (**Figure 7**), which is valuable to keep gene cassettes small. Ugi21 and Ugi22 insulator sequences have been deposited in GenBank as accessions BK063663 and BK063664.

### Conserved motifs among reported insulator-like sequences

**Figure 8:**
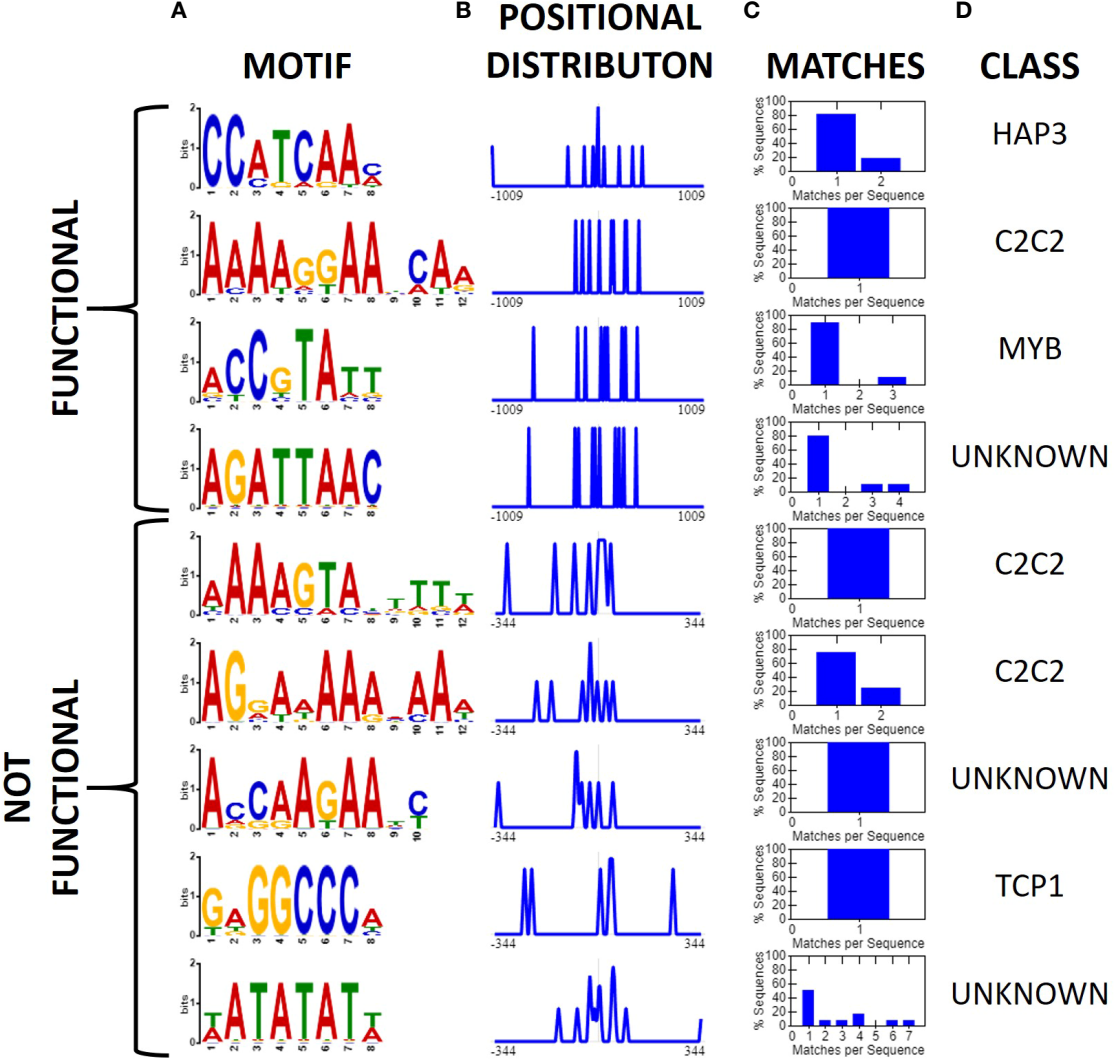
Motifs associated with validated functional and non-functional insulators (XTREME, Bailey et al., 2015). **(A)** Conserved motifs among 12 validated sequences with insulator-like activity in plants (top) or 15 *U. gibba* sequences which showed no insulator-like activity, displayed as a sequence logo, **(B)** positional distribution relative to the middle of the intergenic sequence, and normalized to the maximum, **(C)** Histogram displaying the number of query sequences containing the motif and instances per sequence. **(D)** The putative class of transcription factor or binding site associated with the motif. The C2C2-related 12-nt AAAAGGAABCAA motif is present in all 12 functional insulator sequences, though given the small sample size this should be interpreted with caution. It is also present in other sequences where we could not detect insulator function.

After identifying two new insulator-like sequences, we repeated the earlier motif analysis but limiting our dataset to 12 sequences reported to have insulator-like function in plants: BEAD1c (Zhong and Krangel, 1997), CW198 (Jiang et al., 2022), EXOB (Singer et al., 2010), HIV-LTR (Eggermont and Proudfoot, 1993), RS2-9 (Liu et al., 2022), TBS (Hily et al., 2009), UASrpg (Bi and Broach, 1999), Ugi1, Ugi3, Ugi4 (Illa-Berenguer et al.), Ugi21 and Ugi22 (this study). Pairwise local alignments with BLASTn uncovered no evidence of any conserved regions shared between any pair of reported insulators. Motif search and discovery on the 12 reported insulators identified four putative motifs. Two of the motifs (AAAAGGAABCAA and ACCGTATT) share similarities to the MYB and C2C2 motifs found in the general search. Notably, the C2C2-related motif AAAAGGAABCAA is present in all reported sequences with insulator-like function in plants. In comparison, we applied the same analysis to 15 sequences that did not show insulator-like activity in this assay [eight from this analysis [Ugi25-Ugi38; **Figure 6**] and seven from (Illa-Berenguer et al.)]. In these sequences we found five motifs including two additional “A” rich C2C2 motifs, one TCP related motif and two motifs of unknown class (**Figure 8**). There is no clear evidence of a motif shared by all functional insulators that is not also associated with a promoter. Previous investigations of plant insulator-like elements found a similar pattern (Hily et al., 2009; Singer et al., 2010). However, investigations of the TBS insulator in *Petunia hybrida* showed enhancer-promoter interaction was not exclusively indicated by matrix attachment regions, suggesting that insulator function of this sequence, if indeed present, may be at least partially due to unknown sequence motifs (Hily et al., 2009). Nevertheless, while the 12 nt C2C2 related AAAAGGAABCAA motif is in all reported plant sequences showing insulator function, this motif is also observed in many other sequences that lack insulator function. Therefore, DNA sequence alone is not sufficient to predict insulator function in plants.

## Discussion

In a genetic engineering context, insulator-like elements can reduce cross-regulation within transgenic cassettes, limit enhancer/promoter interference, and protect genes from position-dependent silencing (reviewed in Matzat and Lei, 2014) as well as stabilize transgene expression (Butaye et al., 2005). Thus, the purpose of this study was to mine the *U. gibba* genome for potential sequences that could be used in the creation of multigene cassettes for genetic engineering, with particular focus on sequences with insulator function. Several such sequences have previously been reported, including three other insulators in *U. gibba*: Ugi1 (OK086967.1), Ugi3 (OK086968.1), and Ugi4 (OK086969.1) that were experimentally validated (Illa-Berenguer et al.). All three of these elements were present in our set of *U. gibba* insulators (Ugi1 called as ID2526, Ugi3 called as ID10084 and Ugi4 called as ID4351 in **Supplementary Table 1**). It is notable that all three elements were flanked by genes in the convergent orientation, as were both Ugi21 and Ugi22, suggesting this group may be enriched for more functional elements.

In this study, we attempted to identify unmethylated regions (UMRs) in the genome to enrich for functional sequences with transcription blocking activity, since regulatory sequences are generally located in unmethylated regions (Crisp et al., 2020). Previous analysis of methylation levels in *U. gibba* had shown reduced global methylation levels, likely due to the loss of key genes in the RNA-directed methylation pathway (Cervantes-Pérez et al., 2021). Consequently, we observed large UMRs in the *U. gibba* genome that covered ~87% of our targeted intergenic regions. These UMRs often covered multiple predicted genes and intergenic regions, so that they unfortunately could not be used to narrow the boundaries of sequences with potential insulator like activity in *U. gibba*.

Under the assumption that functional gene sequences are more likely to be conserved, the targeted intergenic regions were also compared to 45 additional physical maps. Only 7.1% of divergent and 10.5% of convergent intergenic regions among our targets were syntenic (i.e., shared gene pairs flanking the potential insulator). The low overall synteny rates may be partly due to incomplete or misassembled genomes for comparison, while the intense genomic shuffling *U. gibba* underwent (Ibarra-Laclette et al., 2013) also makes whole-genome alignments impractical and complicates syntenic analyses. Despite these limitations, both Ugi21 and Ugi22 were identified based on their conserved microsynteny, so the filter appears to have functioned as intended.

After identifying two new insulator-like elements, we searched all 12 plant insulator sequences for shared motifs. A 12 nt C2C2 related AAAAGGAABCAA motif was found in all validated plant transgene insulators; however, since we only have 12 validated sequences to compare, this result must be taken with caution. Pairwise BLASTN comparison of the 12 sequences finds no large-scale sequence similarities (i.e., matches >15 nt). Furthermore, evaluations of insulator-like activity in randomly generated DNA sequences have shown evidence of transcription blocking (Illa-Berenguer et al.). Together this suggests that DNA sequence may be only partially responsible for insulator-like activity. Speculatively, other factors such as tertiary structures may be playing a role, but additional analyses would be required to investigate this. We also found multiple instances of simple sequence repeat motifs among our target regions, but with no discernable pattern. Ultimately, the lack of canonical insulator motifs among our potential insulators may be due to insulators relying on more than just a conserved DNA sequence to function. While these sequences show insulator activity in transgenic constructs, it is important to note that this dataset does not include any evidence that these sequences have insulator-like function in their native context.

There is currently no direct evidence of genomic insulators in plants, and no overlap has been found between insulators across plants and animals. This may indicate either that the last common ancestor of the two groups lacked chromatin insulators or that plants have lost them since divergence (Liu et al., 2017). Orthologs of known animal insulator binding proteins such as Rap2, CTCF, and Su(Hw) have not yet been identified in plant genomes (Liu and Weigel, 2015; Kurbidaeva and Purugganan, 2021).

The assumption that genomic insulators should exist in plants comes from insulators being identified in both animals and fungi, suggesting some conservation of genome organizational structure among eukaryotes (Akasaka et al., 1999; Scott et al., 2006; Heger and Wiehe, 2014). In support of this argument, DNA-binding domains of other species’ insulator proteins (Rap1, Su(Hw), and CTCF) expressed in *Arabidopsis* showed context-dependent genomic insulator activity (Tran and Johnson, 2020).

Our study shows effective insulator activity in two newly found sequences. Additional insulator-like sequences likely remain to be found among our candidates. For example, several potential insulator sequences from this work could not be synthesized or cloned despite repeated attempts, so they could not be tested for validation. Furthermore, the elements tested in this study were only tested in one orientation; it is possible that some of the sequences may function when placed in the opposite orientation. The full-length TBS and Ugi3 activity is orientation-dependent, being less effective in reverse orientation (Hily et al., 2009; Illa-Berenguer et al.), whereas Ugi1 and Ugi4 showed no activity when reversed (Illa-Berenguer et al.). Furthermore, no insulator activity was observed when the full-length UASrpg and BEAD1c sequences were tested in our transgenic context (Illa-Berenguer et al.).

Finally, this study probes the structure and conservation of the highly compact *Utricularia gibba* genome and presents an atlas of intergenic regions with the potential for insulator activity. The lack of understanding of plant insulator-like function represents a significant challenge moving forward, but the identification of two additional *U. gibba* sequences with validated insulator function in a transgenic context and the fact that all five known *U. gibba* insulators are between convergent genes provide a solid basis on which to conduct further investigations. These findings add two insulator sequences to the pool of *Utricularia* sequences able to function in plants in our assays, namely, Ugi1, Ugi3, Ugi4 (Illa-Berenguer et al.), RS2-9 (Liu et al., 2022) and EXOB (Singer et al., 2010). Furthermore, Ugi22 represents the shortest functional insulator sequence among the seven with a length of only 390 nt.The fact that including this work, five insulator-like sequences were uncovered in *Utricularia gibba* is remarkable given only two were reported in the considerably larger *Oryza sativa* genome (Zhang et al., 2012; Liu et al., 2022; Illa-Berenguer et al.) indicates the value of mining compact genomes.

## Methods

### RNAseq mapping and identification of potential regulatory sequences

Raw *Utricularia gibba* RNAseq data were downloaded from NCBI (bioprojects PRJNA595351 and PRJNA354080). These datasets consist of single-end reads for bladder, leaf, stem, and rhizoid (root-like) tissue, plus paired-end reads for pooled vegetative tissue, shoots, and traps. Reads were trimmed with Trimmomatic (Bolger et al., 2014) (ILLUMINACLIP, max mismatch 2, palindrome clip 30, match accuracy 10, SLIDINGWINDOW, 5nt window length, minimum quality 20, MINLEN minimum read length 36nt). The trimmed reads were quality checked with FastQC (Andrews, 2015) to verify adapter removal and data quality. All reads were mapped to the *U. gibba* PacBio physical map (Lan et al., 2017) using TopHat2 (Kim et al., 2013) (-N 1, -g 1). Paired reads were pooled and mapped as pairs, while single-end reads pairs, where one mate was lost during trimming, were pooled and mapped as a separate dataset with the same command. The resulting BAM alignment file was analyzed with Cufflinks (Trapnell et al., 2012) to identify potential gene models. Cufflinks identified 30,756 transcripts, compared to 30,689 reported in the published PacBio Assembly.

Sequences with potential insulator like activity were classified based on read depth (FPKM, fragments per kilobase per million reads) and relative gene orientation of the neighboring genes. For read depth, potential insulators were required to have an FPKM fold change between gene models >= 1.5 with the intent to categorize as many potential CRE regions as possible. We also required the higher expressed model to be in the top 50% of transcripts. The gene models also had to be non-overlapping and less than 20 kb apart to account for up to ~2 retrotransposon insertions.

Divergent intergenic regions with potential insulator activity were called when genes were diverging and either gene was higher expressed than the other. Convergent intergenic regions with potential insulator activity were called as convergent gene pairs with only one gene highly expressed. See **Figure 2**, **Supplementary Figure 4** for a graphical representation of these transcriptional profiles.

### Identifying syntenic potential regulatory sequences

To identify syntenic regions, the translated protein sequence of the genes flanking sequences with potential insulator function were extracted and aligned to a database of protein sequences for each of the 44 genomes using BLASTP (Camacho et al., 2009) (E-value<10−5 without filtering for low-complexity sequences). The resulting blast output was parsed to identify orthologous pairs, which were defined as those where a) both genes have BLASTP hits in the ortholog and b) there are ≤ 5 genes between the gene pairs. The orthologous gene pairs were scanned to identify syntenic groups of potential sequences with insulator function. Synteny was classified based on the orientation and number of gene models in *U. gibba* compared to the orthologs. If the orientation between the pair and the number of gene models in the region was conserved in *U. gibba* and the orthologous species, it was called as a high-quality microsyntenic region. If the orientation was conserved and the number of gene models differ between *U. gibba* and the ortholog it was called as a deletion/insertion. Finally, if the orientation of one of the genes in a pair in either *U. gibba* or the ortholog differs but the number of gene models in the region was conserved it was called as a potential inversion. A presence-absence table was created from these data to evaluate the prevalence of potential regulatory regions identified in *U. gibba.*

### Mapping whole genome bisulfite sequencing data and calling UMRs

Raw bisulfite-seq reads were downloaded from NCBI (accessions SRR12007530 & SRR12007531) (Cervantes-Pérez et al., 2021) and trimmed using Cutadapt v3.4 (Martin, 2011). The trimmed reads were then mapped to the *U. gibba* physical map with Bowtie2 (Langmead and Salzberg, 2012). The resulting BAM file was screened with Picard (https://broadinstitute.github.io/picard/command-line-overview.html) to remove duplicate reads. Methylation levels were then quantified with Methylpy (Schultz et al., 2015) in 100-bp non-overlapping windows over the genome. The 100-bp tiles with fewer than two cytosines and less than 5x coverage were defined as missing data as they do not provide enough methylation data for analysis. The tiles with less than 10% methylation in the CG, CHG, and CHH contexts were called as unmethylated (Crisp et al., 2020). The tiles that are defined as missing data and unmethylated were merged. Merged tiles with no more than 33% missing data and covering at least 300 bp are defined as unmethylated regions. A flowchart with further details on these methods can be found in **Supplementary Figure 5**.

### Insulator motif analysis

The potential insulators and ambiguous terminator/insulators were searched for motifs using XSTREME as part of the MEME suite web server (https://meme-suite.org/meme/index.html, (Bailey et al., 2015). XSTREME was used to discover and identify enriched motifs in the potential insulators the ArabidopsisDAPv1 was used as a reference to search for known plant motifs (xstreme –oc. –time 240 –streme-totallength 4000000 –meme-searchsize 100000 –fdesc description –dna –evt 0.05 –minw 6 –maxw 15 –align center –meme-mod zoops –m db/motif_databases/ARABD/ArabidopsisDAPv1.meme –p PUTATIVE_INSULATORS.fa). For more information see **Supplemental File Image 1.PNG**.

### Selecting candidates for *in vivo* validation

Candidates were selected by sorting the potential insulators based on fold change alone. The nine highest candidates were selected from this group regardless of whether they were syntenic. To extract the syntenic insulators we sorted the list based on those that had orthologs in the largest number of genomes regardless of fold change. The nine candidates shared with the largest number of genomes were tested for validation. In both cases potential insulators were selected regardless of whether they were convergent or divergent. These fragments were ordered using preliminary data from the characterization pipeline for our intergenic regions resulting in differences between the validated sequences and their counterparts in the synteny data of **Supplementary Table 1**.

### Evaluation of potential insulator activity in *N. benthamiana*

Ten bladderwort sequences (**Supplementary Table 1**) were evaluated for their use as potential insulators as described by (Illa-Berenguer et al.). Four of these sequences were identified based on fold-change difference in expression levels and the 6 additional ones based on syntenic criteria, as described in Identifying Syntenic Potential Regulatory Sequences section of Material and Methods. These sequences were synthesized as fragments by Azenta Life Sciences (South Plainfield, NJ, US) and Integrated DNA Technologies, Inc. (Coralville, IA, USA) and cloned using the GreenGate cloning system (Lampropoulos et al., 2013) as described by (Illa-Berenguer et al.).

*Agrobacterium tumefaciens* strain LBA4404 (Hoekema et al., 1983) was used for transient transformation as described by (Illa-Berenguer et al.) with the following differences. Twenty-five µL of electrocompetent LBA4404 cells were electroporated in a Bio-Rad MicroPulser™ electroporator (BioRad Laboratories, Hercules CA, US) according to the manufacturer’s user guide with 1 µL of assembled plasmid prep. Colonies containing the binary vectors were grown at 28°C on YM medium plates supplemented with 100 µg · mL^-1^ streptomycin for selection of the *Agrobacterium* strain and 100 µg · mL^-1^ spectinomycin for selection of the binary vectors. Isolated single colonies were inoculated to YM liquid medium complemented both streptomycin and spectinomycin and incubated with continuous agitation at 240 rpm at 28°C. The overnight culture was scaled up 50-fold in 100 mL baffled flask containing 25 mL of YM liquid medium with same antibiotic concentration. Cell cultures were pelleted by centrifugation at 3500 ×g for 15 minutes and resuspended in infiltration buffer (10 mM MgCl_2_, 2-(N-morpholino)ethanesulfonic acid (MES) (pH 5.6), 100 mM acetosyringone) to OD_600_ of 0.6-0.7. Finally, *Agrobacterium* cells were incubated at 21°C for 3 hours prior agroinfiltration.

Three leaves of 4-week-old *N. benthamiana* TW17 plants were infiltrated through the abaxial surface with a BD™ 1-mL TB needleless syringe (Becton, Dickinson and Company, Franklin Lakes, NJ, USA). After infiltration, plants were kept in growth room for 3 days at 23°C with 15 h/9 h day/night photoperiod and light intensity of 200 μmol m^-2^ s^-1^.

Fluorescence intensity measurements were performed as previously described (Pasin et al., 2014). *N. benthamina* leaf discs were obtained with a 6-mm diameter disposable biopsy punch (Sklar Corporation, West Chester, PA, USA) and floated adaxial side down in the wells of a black flat-bottom 96-well plate (Caplugs Evergreen, Buffalo, NY, USA) with 200 µL of water. Top reading measurements were taken using a Biotek SynergyTM 2 plate reader (BioTek Instruments Inc., Winooski, VT, USA) with BioTek Gen5 Microplate Data Collection software version 3.09 (BioTek Instruments Inc., Winooski, VT, USA). GFP values were collected through a 485/20 nm excitation filter, 510 nm dichroic mirror and 516/20 nm emission filter. Acquisition settings for mCherry were 585/10 nm excitation filter, 595 nm dichroic mirror and 620/15 nm emission filter. Gain value was adjusted manually at 50 to avoid signal saturation.

Insulator activity was inferred from the difference between the GFP and mCherry measurements (as GFP:mCherry ratio). In addition, fold change (FC) differences between the potential insulator sequences and the minimal sequence (21 bp) that can separate the two cassettes were calculated as


$$FC=\frac{\left(\frac{GFP_{i}}{mCherry_{i}}\right)-\left(\frac{GFP_{21bp\;spacer}}{mCherry_{21bp\;spacer}}\right)}{\left(\frac{GFP_{21bp\;spacer}}{mCherry_{21bp\;spacer}}\right)}$$

to evaluate the impact of the DNA fragment size on insulator activity.

## Data availability statement

Publicly available datasets were analyzed in this study. This data can be found here: NCBI Bioprojects PRJNA595351, PRJNA354080, PRJNA633566, and PRJNA383049.

## Author contributions

DL: Data curation, Formal Analysis, Investigation, Methodology, Project administration, Software, Visualization, Writing – original draft, Writing – review & editing. EI: Data curation, Formal Analysis, Investigation, Methodology, Software, Validation, Visualization, Writing – original draft, Writing – review & editing. SB: Data curation, Formal Analysis, Investigation, Methodology, Software, Visualization, Writing – original draft, Writing – review & editing. RS: Investigation, Resources, Supervision, Writing – review & editing. WP: Conceptualization, Funding acquisition, Methodology, Project administration, Resources, Supervision, Validation, Writing – review & editing. JW: Conceptualization, Funding acquisition, Methodology, Project administration, Resources, Supervision, Writing – review & editing, Investigation.
