# Corrigendum: Mining the *Utricularia gibba* genome for insulator-like elements for genetic engineering

**DOI:** 10.3389/fpls.2024.1412239

**Published:** 2024-04-26

**Authors:** Daniel Laspisa, Eudald Illa-Berenguer, Sohyun Bang, Robert J. Schmitz, Wayne Parrott, Jason Wallace

**Affiliations:** ^1^ Center for Applied Genetic Technologies, University of Georgia, Athens, GA, United States; ^2^ Institute of Bioinformatics, University of Georgia, Athens, GA, United States; ^3^ Department of Genetics, University of Georgia, Athens, GA, United States; ^4^ Department of Crop & Soil Science & Institute of Plant Breeding, Genetics and Genomics, University of Georgia, Athens, GA, United States

**Keywords:** bladderwort, insulator, transgenics, *Utricularia*, *cis*-regulatory elements

In the published article, an author name was incorrectly written as Eudald llla-Berenguer. The correct spelling is Eudald Illa-Berenguer.

The authors apologize for this error and state that this does not change the scientific conclusions of the article in any way. The original article has been updated.

