# Supplementary figures and images for: Mining the Utricularia gibba genome for insulator-like elements for genetic engineering
Source: Front Plant Sci. 2023 Nov 8;14:1279231. doi: 10.3389/fpls.2023.1279231 (PMC10663240; doi:10.3389/fpls.2023.1279231)

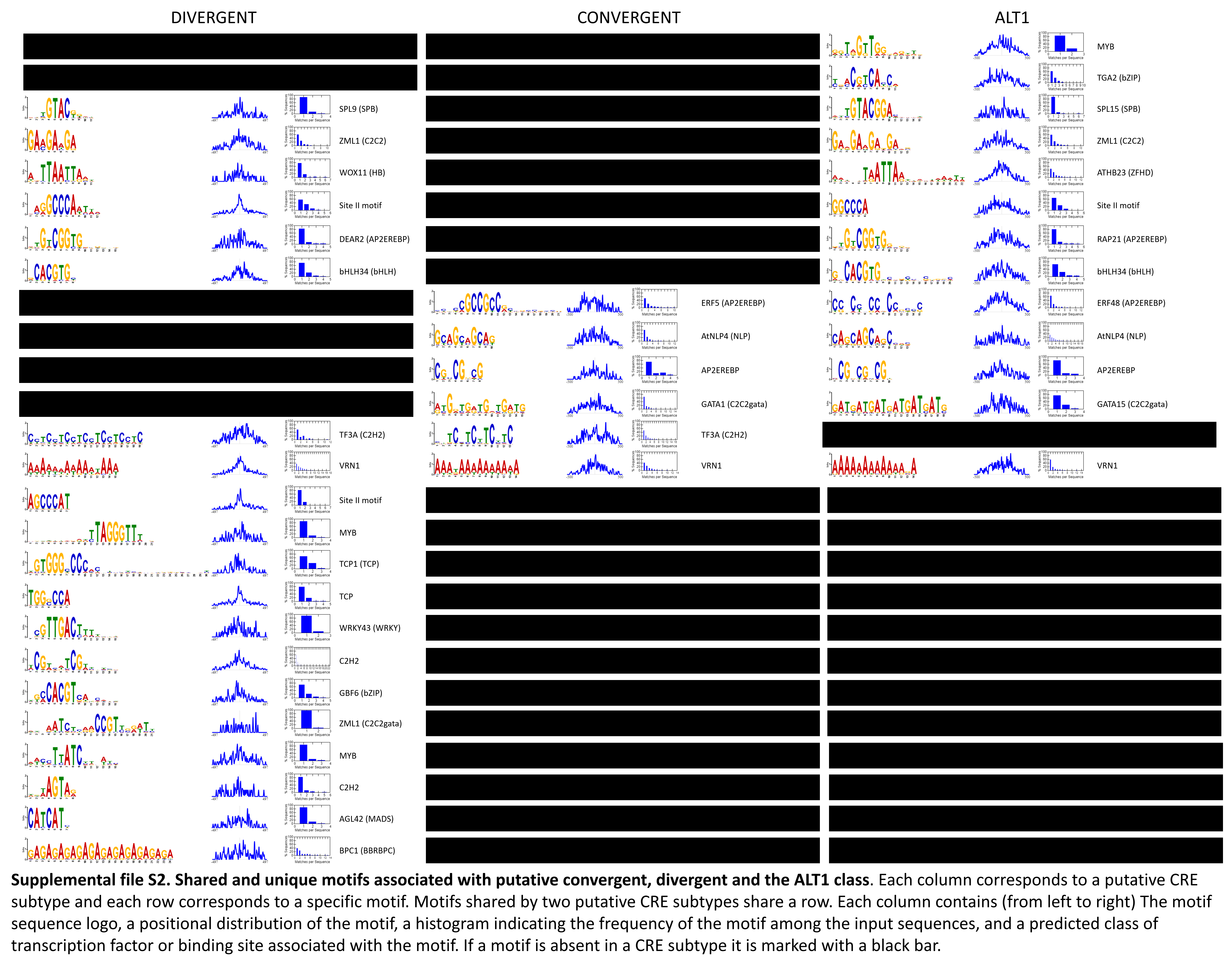

Supplement: Supplementary file 1 [file Image_1.png]
