# Supplementary material for: Mining the Utricularia gibba genome for insulator-like elements for genetic engineering
Source: Front Plant Sci. 2023 Nov 8;14:1279231. doi: 10.3389/fpls.2023.1279231 (PMC10663240; doi:10.3389/fpls.2023.1279231)
Supplement: Supplementary file 2 [file Presentation_1.pptx]

## Slide 1
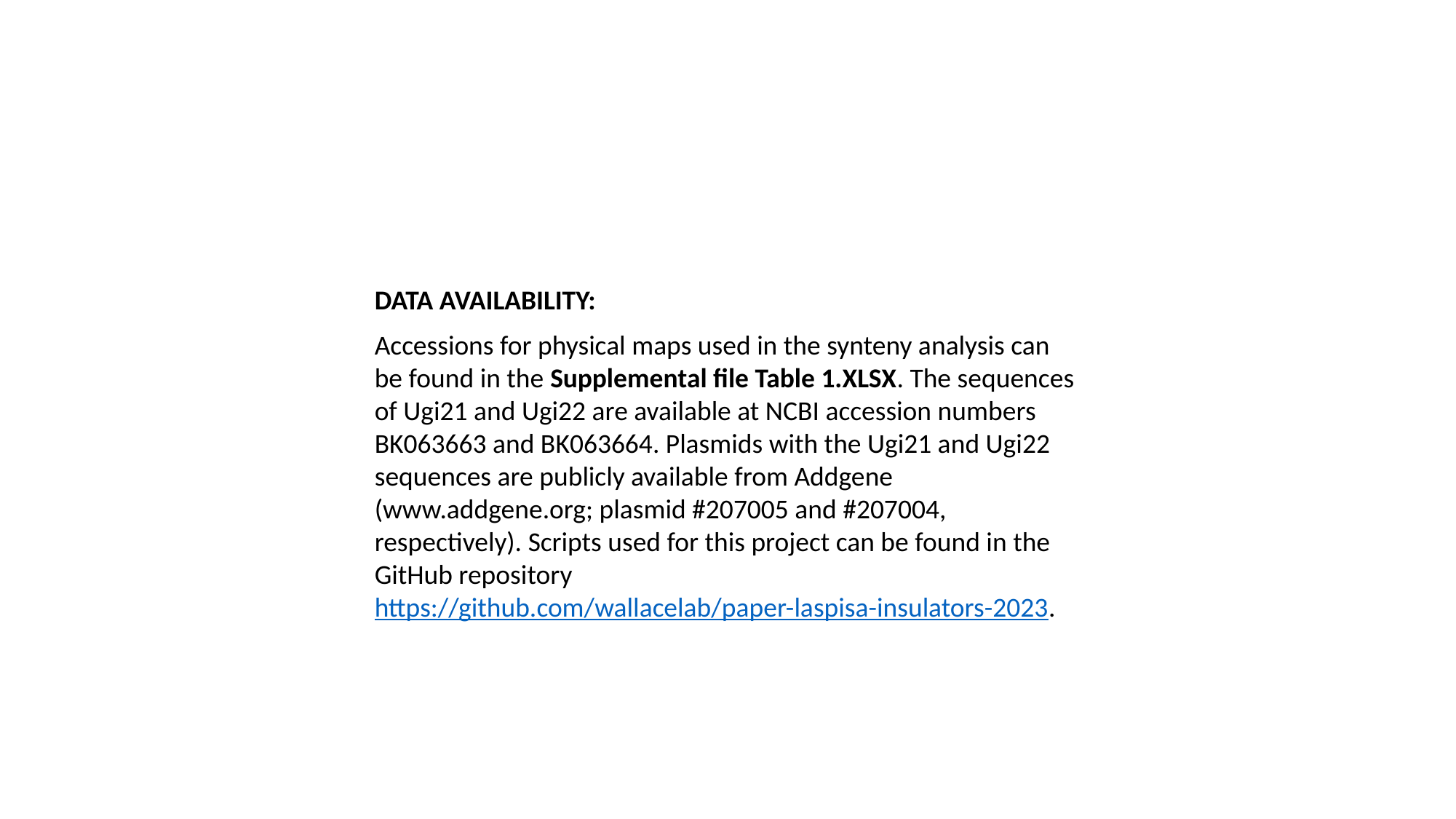

DATA AVAILABILITY:
Accessions for physical maps used in the synteny analysis can be found in the Supplemental file Table 1.XLSX. The sequences of Ugi21 and Ugi22 are available at NCBI accession numbers BK063663 and BK063664. Plasmids with the Ugi21 and Ugi22 sequences are publicly available from Addgene (www.addgene.org; plasmid #207005 and #207004, respectively). Scripts used for this project can be found in the GitHub repository https://github.com/wallacelab/paper-laspisa-insulators-2023.

## Slide 2
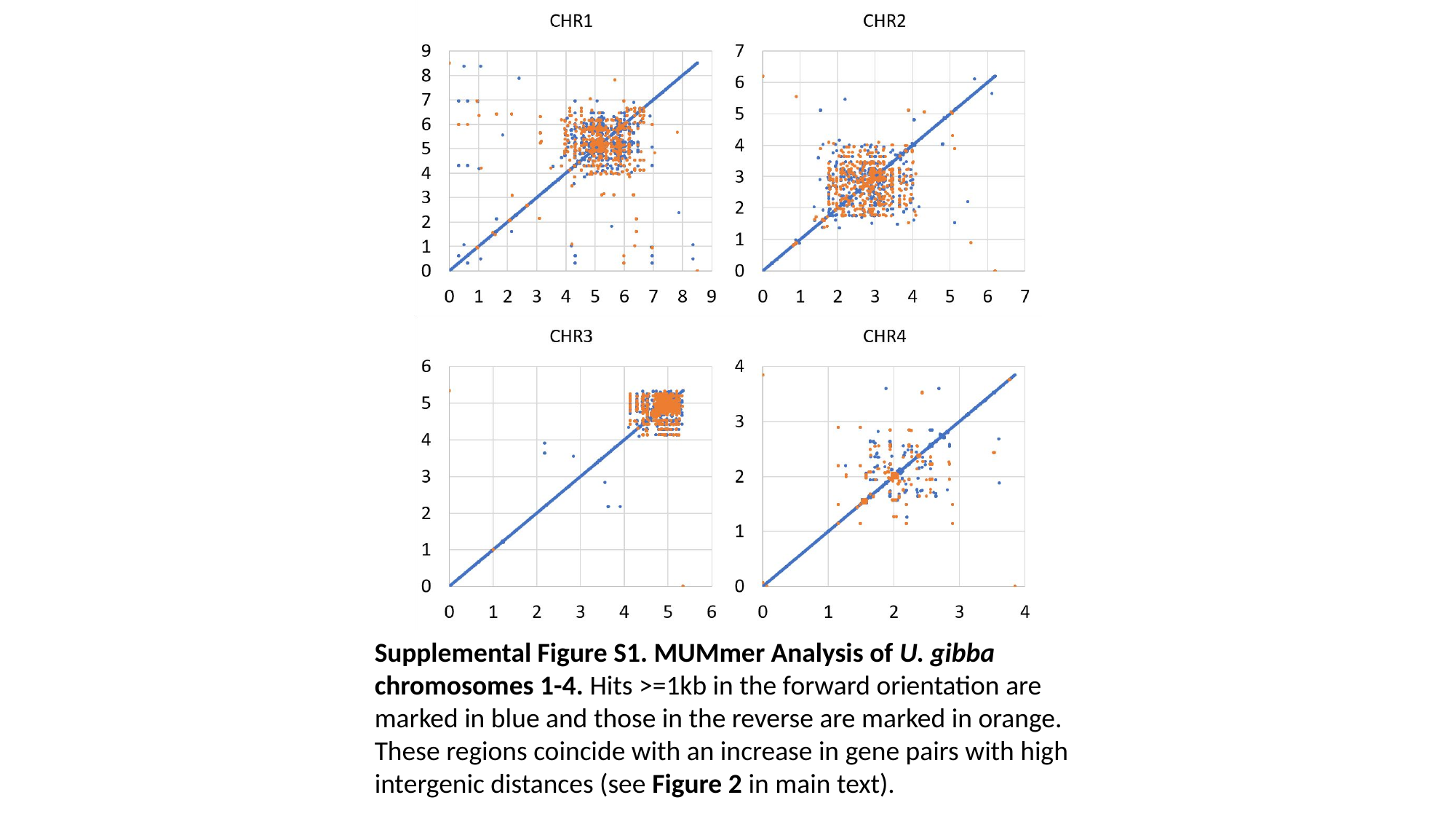

Supplemental Figure S1. MUMmer Analysis of U. gibba chromosomes 1-4. Hits >=1kb in the forward orientation are marked in blue and those in the reverse are marked in orange. These regions coincide with an increase in gene pairs with high intergenic distances (see Figure 2 in main text).

## Slide 3
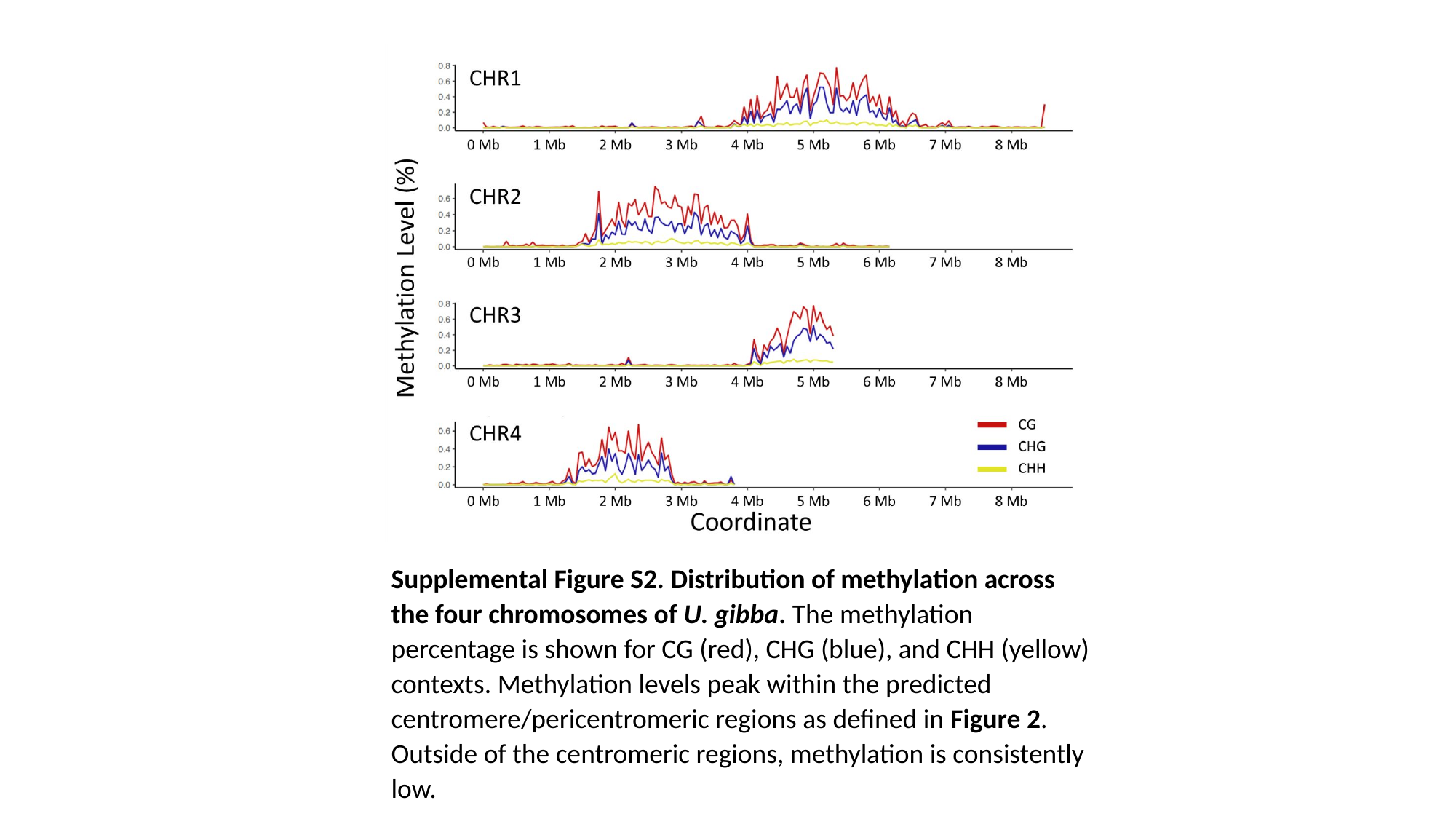

Supplemental Figure S2. Distribution of methylation across the four chromosomes of U. gibba. The methylation percentage is shown for CG (red), CHG (blue), and CHH (yellow) contexts. Methylation levels peak within the predicted centromere/pericentromeric regions as defined in Figure 2. Outside of the centromeric regions, methylation is consistently low.

## Slide 4
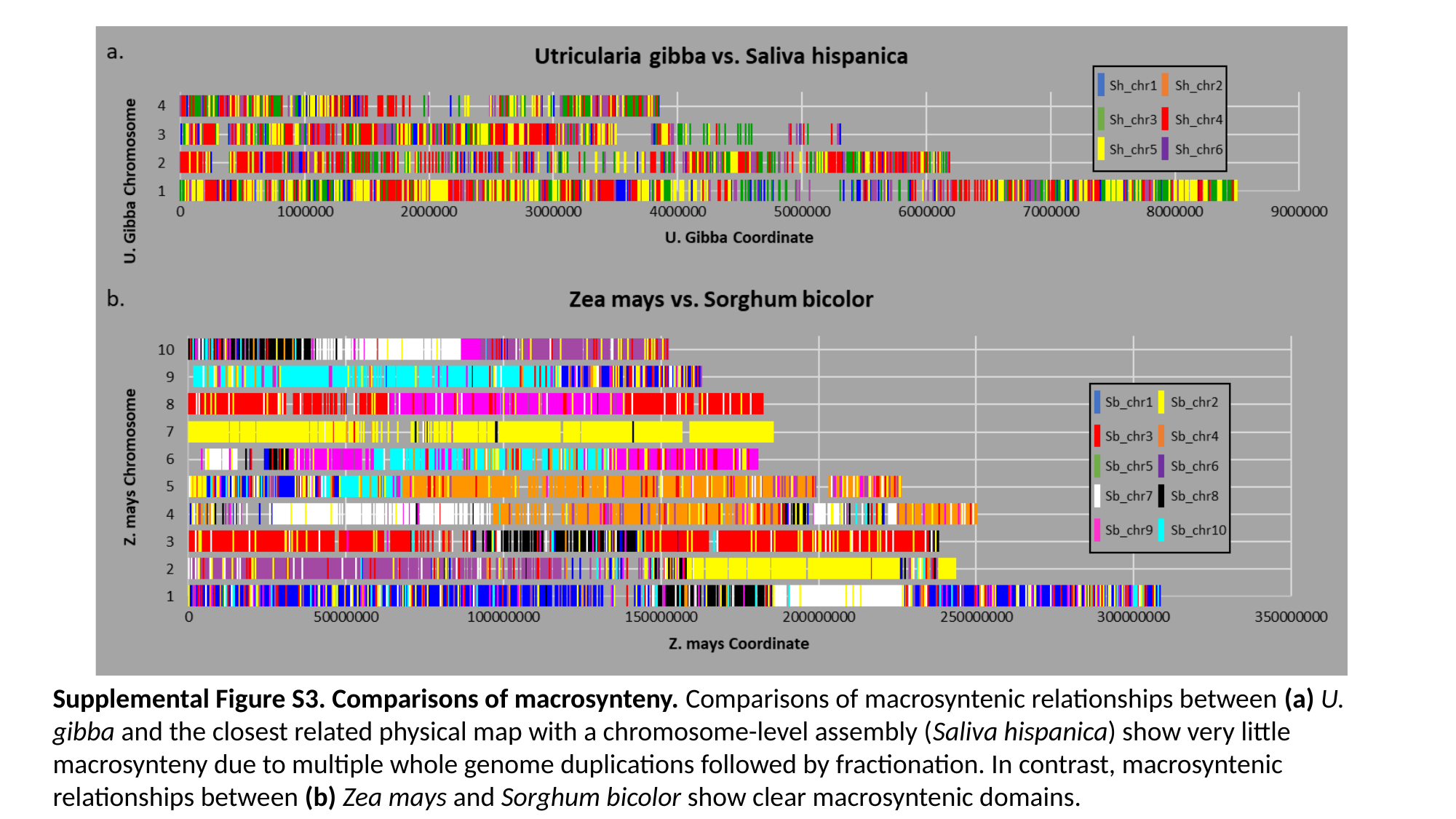

Supplemental Figure S3. Comparisons of macrosynteny. Comparisons of macrosyntenic relationships between (a) U. gibba and the closest related physical map with a chromosome-level assembly (Saliva hispanica) show very little macrosynteny due to multiple whole genome duplications followed by fractionation. In contrast, macrosyntenic relationships between (b) Zea mays and Sorghum bicolor show clear macrosyntenic domains.

## Slide 5
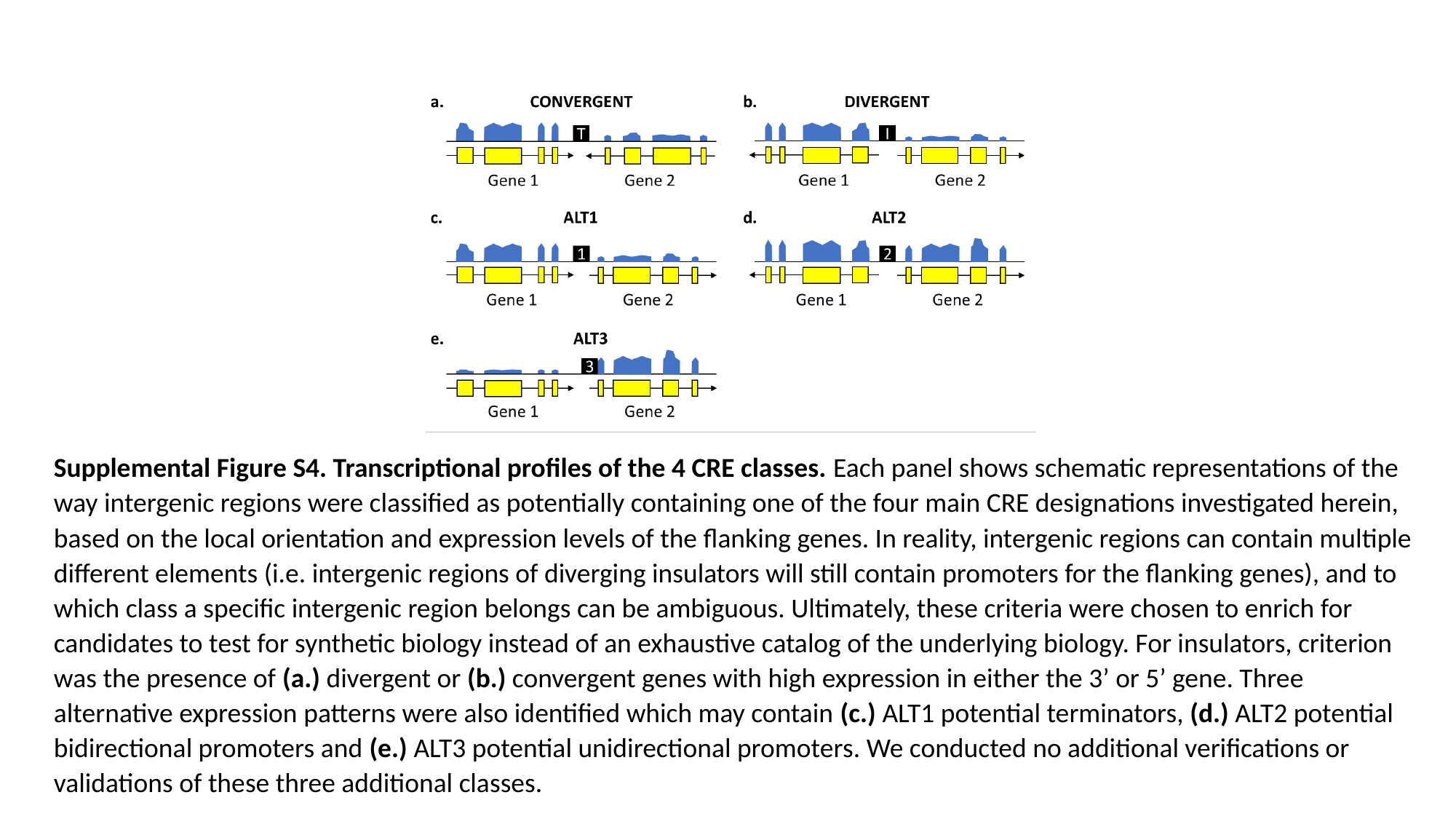

Supplemental Figure S4. Transcriptional profiles of the 4 CRE classes. Each panel shows schematic representations of the way intergenic regions were classified as potentially containing one of the four main CRE designations investigated herein, based on the local orientation and expression levels of the flanking genes. In reality, intergenic regions can contain multiple different elements (i.e. intergenic regions of diverging insulators will still contain promoters for the flanking genes), and to which class a specific intergenic region belongs can be ambiguous. Ultimately, these criteria were chosen to enrich for candidates to test for synthetic biology instead of an exhaustive catalog of the underlying biology. For insulators, criterion was the presence of (a.) divergent or (b.) convergent genes with high expression in either the 3’ or 5’ gene. Three alternative expression patterns were also identified which may contain (c.) ALT1 potential terminators, (d.) ALT2 potential bidirectional promoters and (e.) ALT3 potential unidirectional promoters. We conducted no additional verifications or validations of these three additional classes.

## Slide 6
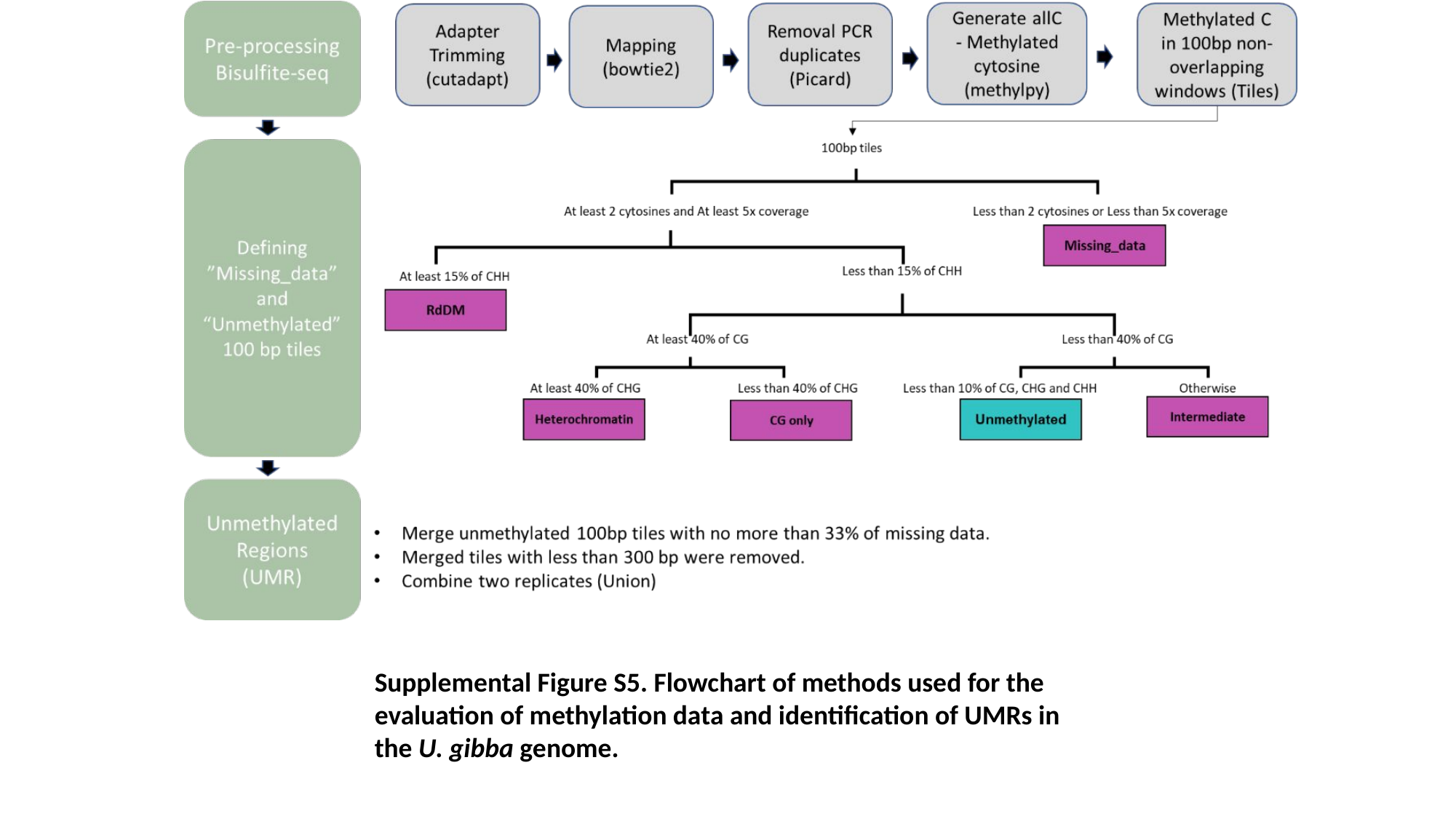

Supplemental Figure S5. Flowchart of methods used for the evaluation of methylation data and identification of UMRs in the U. gibba genome.

## Slide 7
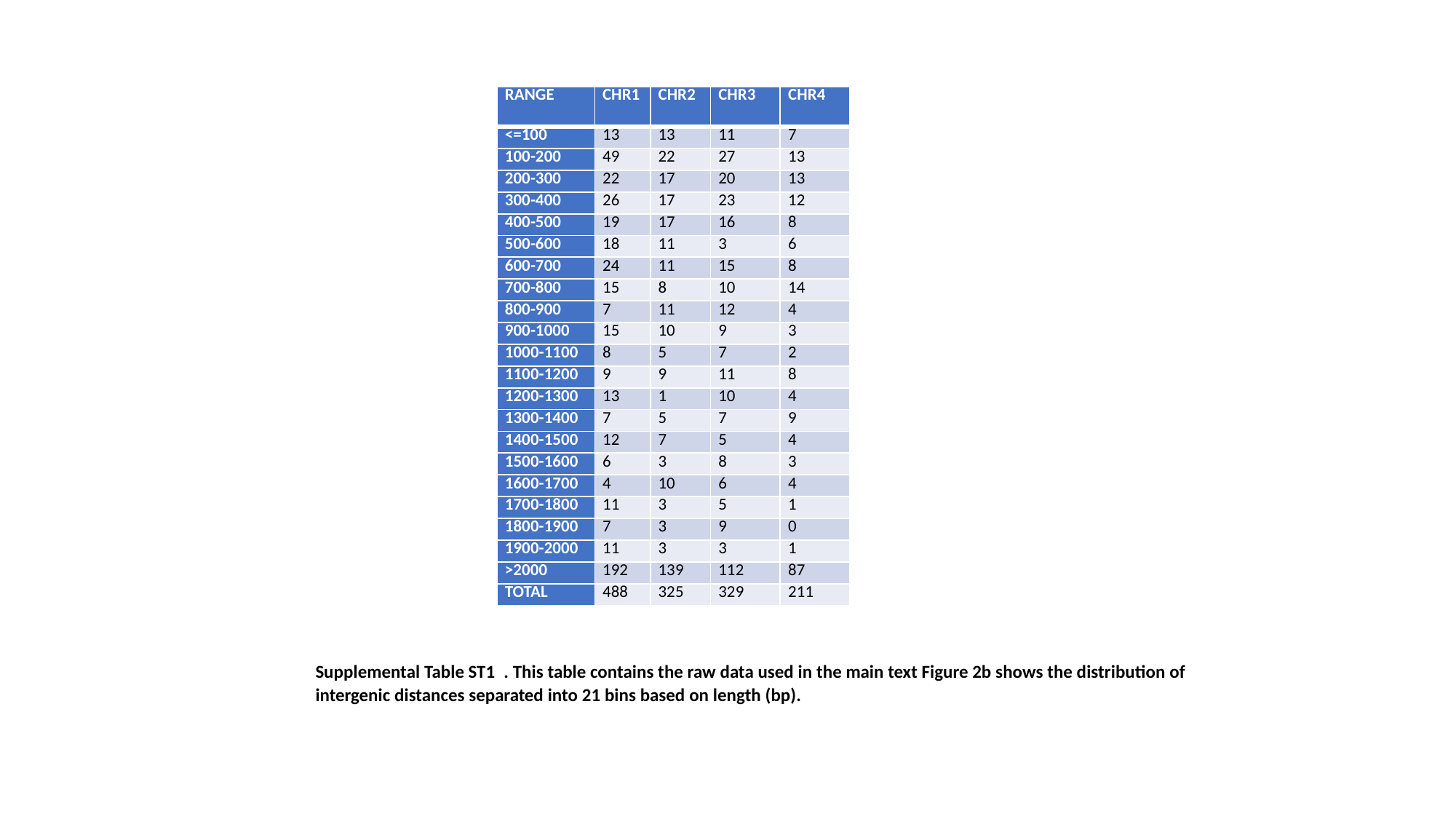

| RANGE | CHR1 | CHR2 | CHR3 | CHR4 |
| --- | --- | --- | --- | --- |
| <=100 | 13 | 13 | 11 | 7 |
| 100-200 | 49 | 22 | 27 | 13 |
| 200-300 | 22 | 17 | 20 | 13 |
| 300-400 | 26 | 17 | 23 | 12 |
| 400-500 | 19 | 17 | 16 | 8 |
| 500-600 | 18 | 11 | 3 | 6 |
| 600-700 | 24 | 11 | 15 | 8 |
| 700-800 | 15 | 8 | 10 | 14 |
| 800-900 | 7 | 11 | 12 | 4 |
| 900-1000 | 15 | 10 | 9 | 3 |
| 1000-1100 | 8 | 5 | 7 | 2 |
| 1100-1200 | 9 | 9 | 11 | 8 |
| 1200-1300 | 13 | 1 | 10 | 4 |
| 1300-1400 | 7 | 5 | 7 | 9 |
| 1400-1500 | 12 | 7 | 5 | 4 |
| 1500-1600 | 6 | 3 | 8 | 3 |
| 1600-1700 | 4 | 10 | 6 | 4 |
| 1700-1800 | 11 | 3 | 5 | 1 |
| 1800-1900 | 7 | 3 | 9 | 0 |
| 1900-2000 | 11 | 3 | 3 | 1 |
| >2000 | 192 | 139 | 112 | 87 |
| TOTAL | 488 | 325 | 329 | 211 |
Supplemental Table ST1  . This table contains the raw data used in the main text Figure 2b shows the distribution of intergenic distances separated into 21 bins based on length (bp).

## Slide 8
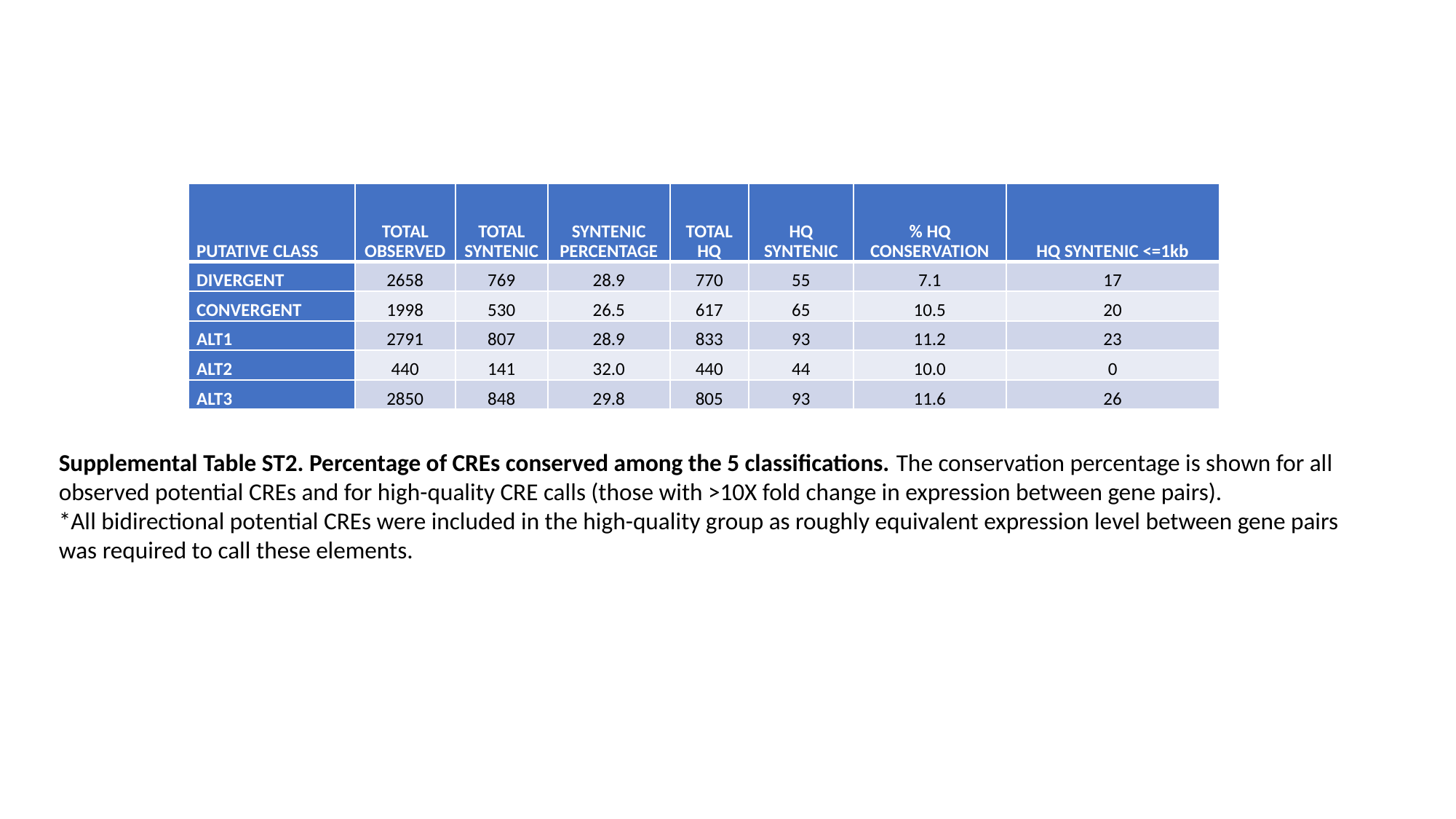

| PUTATIVE CLASS | TOTAL OBSERVED | TOTAL SYNTENIC | SYNTENIC PERCENTAGE | TOTAL HQ | HQ SYNTENIC | % HQ CONSERVATION | HQ SYNTENIC <=1kb |
| --- | --- | --- | --- | --- | --- | --- | --- |
| DIVERGENT | 2658 | 769 | 28.9 | 770 | 55 | 7.1 | 17 |
| CONVERGENT | 1998 | 530 | 26.5 | 617 | 65 | 10.5 | 20 |
| ALT1 | 2791 | 807 | 28.9 | 833 | 93 | 11.2 | 23 |
| ALT2 | 440 | 141 | 32.0 | 440 | 44 | 10.0 | 0 |
| ALT3 | 2850 | 848 | 29.8 | 805 | 93 | 11.6 | 26 |
Supplemental Table ST2. Percentage of CREs conserved among the 5 classifications. The conservation percentage is shown for all observed potential CREs and for high-quality CRE calls (those with >10X fold change in expression between gene pairs).
*All bidirectional potential CREs were included in the high-quality group as roughly equivalent expression level between gene pairs was required to call these elements.
